# Resonant inelastic X-ray scattering endstation at the 1C beamline of Pohang Light Source II

**DOI:** 10.1107/S1600577523001625

**Published:** 2023-03-22

**Authors:** Jin-Kwang Kim, Christopher Dietl, Hyun-Woo J. Kim, Seung-Hyeok Ha, Jimin Kim, Ayman H. Said, Jungho Kim, B. J. Kim

**Affiliations:** a Department of Physics, Pohang University of Science and Technology, Pohang 790-784, Republic of Korea; b Center for Artificial Low Dimensional Electronic Systems, Institute for Basic Science (IBS), 77 Cheongam-Ro, Pohang 37673, Republic of Korea; c Advanced Photon Source, Argonne National Laboratory, Lemont, IL 60439, USA; ESRF – The European Synchrotron, France

**Keywords:** resonant inelastic X-ray scattering, RIXS spectrometer, 1C beamline, PLS-II, hard X-rays

## Abstract

An endstation for resonant inelastic X-ray scattering (RIXS), dedicated to operations in the hard X-ray regime, has been constructed at the 1C beamline of Pohang Light Source II. At the Ir *L*_3_-edge, a total energy resolution of 34.2 meV was achieved, close to the theoretical estimation of 34.0 meV, which considers factors such as the incident energy bandpass, intrinsic analyzer resolution, geometrical broadening of the spectrometer, finite beam-size effect and Johann aberration. The performance of the RIXS instrument is demonstrated by measuring the RIXS spectra of Sr_2_IrO_4_. The endstation can be easily reconfigured to measure energy-integrated intensities with very low background for diffuse scattering and diffraction experiments.

## Abstract 2

A hard X-ray resonant inelastic X-ray scattering endstation has been developed and commissioned at the 1C beamline of Pohang Light Source II.

## 1. Introduction

Resonant inelastic X-ray scattering (RIXS) has emerged in the last decade as a complementary tool to inelastic neutron scattering for measuring momentum- and energy-resolved excitation spectra of magnetic materials, since the observations of two-magnon (Hill *et al.*, 2008 ▸) and single-magnon (Braicovich *et al.*, 2009 ▸) excitations using RIXS at the Cu *K*-edge (8.99 keV) and *L*_3_-edge (930 eV), respectively. In the hard X-ray regime, single-magnon excitations were first revealed in Sr_2_IrO_4_ (Kim *et al.*, 2012*a* ▸) using a medium-energy-resolution RIXS instrument (Shvyd’ko *et al.*, 2013 ▸). An energy resolution better than 100 meV (Shvyd’ko *et al.*, 2013 ▸; Gog *et al.*, 2013 ▸; Moretti Sala *et al.*, 2018 ▸) allowed low-energy features in an excitation spectrum to be resolved, including single-magnon excitations. The hard X-ray RIXS spectrometer is based on the Rowland circle geometry in a near-backscattering condition which, with a position-sensitive detector and an improved energy bandpass of the incident X-rays, has seen a dramatic enhancement in the energy resolution and the photon counts (Shvyd’ko *et al.*, 2013 ▸; Huotari *et al.*, 2005 ▸). Now that the theoretical energy resolution based on this scheme has been achieved, the development of a new type of spectrometer using flat crystal optics is underway (Kim *et al.*, 2018 ▸), which is also advantageous for high-pressure experiments (Kim *et al.*, 2020 ▸).

RIXS is a photon-in photon-out spectroscopy which can, in principle, analyse all kinds of bosonic excitations from charge, orbital, spin and lattice degrees of freedom by measuring the changes in energy, momentum (Ament *et al.*, 2011*a* ▸) and polarization of the scattered photons (Gao *et al.*, 2016 ▸; Ishii *et al.*, 2013 ▸). Resonant processes in RIXS can enhance the sensitivities to low-energy excitations of specific elements and orbitals, which are selected by tuning the incident X-ray energies to an absorption edge of an element in the sample. Advances in the theoretical interpretation of the RIXS process have been made in parallel (Haverkort, 2010 ▸; Ament *et al.*, 2011*b* ▸; Kim & Khaliullin, 2017 ▸) and RIXS is now considered a major tool for studying magnetic and electronic excitations of strongly correlated electron systems. For example, RIXS has been applied to studies of the magnetism of the single-layer square-lattice iridate Sr_2_IrO_4_ (Kim *et al.*, 2012*a* ▸), its bilayer counterpart Sr_3_Ir_2_O_7_ (Kim *et al.*, 2012*b* ▸; Moretti Sala *et al.*, 2015 ▸) and honeycomb iridates (Chun *et al.*, 2015 ▸).

In this paper, we report the successful commissioning of the RIXS endstation at the 1C beamline of Pohang Light Source II (PLS-II). We describe the main components of the endstation, which include monochromators, focusing mirrors and a spectrometer, and show their characteristics and performances. We demonstrate the performance of our RIXS instrument tested by acquiring low-energy excitation spectra of Sr_2_IrO_4_. We also demonstrate efficient measurement of X-ray diffuse scattering images by slightly reconfiguring the setup.

## 2. Beamline overview

**Figure 1:**
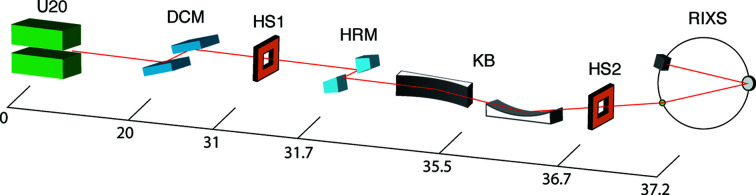
Schematic of the layout for the RIXS endstation at the 1C beamline. The beam propagates from left to right and the position of the beamline components with respect to the center of the undulator are given in meters.

Fig. 1 ▸ shows the schematic layout of the 1C beamline with the components relevant to the RIXS instrument. The source (U20) consists of a 20 mm-period in-vacuum Sm_2_Co_17_ undulator (total 70 periods, 1.4 m long) with an average effective peak field of 0.81 T at a 5 mm gap. The first major optical component is the nitrogen-cooled Si(111) double-crystal monochromator (DCM), which provides the accessible energy range 2.3 to 23 keV. The vertical position of the second crystal is adjusted when changing the energy to fix the height of the exit beam. For RIXS at the Ir *L*_3_-edge (11.215 keV), the DCM provides X-rays with a bandwidth around 1 eV using the fifth-order harmonic. After the DCM, the beam is defined in real space by a four-blade slit (HS1) before entering the experimental hutch. A high-resolution monochromator (HRM), a single channel-cut, is inserted to further narrow the bandwidth. For the Ir *L*_3_-edge, an Si(844) channel-cut is used to narrow the energy bandwidth down to ∼15.8 meV (Gog *et al.*, 2013 ▸). A pair of Kirkpatrick–Baez (KB) mirrors (Kirkpatrick & Baez, 1948 ▸) followed by another four-blade slit (HS2) that defines the beam position after reflections then focus the monochromatic beam on the sample position. The sample is mounted on a closed-cycle helium cryostat which covers the temperature range 4 K to 350 K. The energies and momenta of scattered photons from the sample are analyzed by a RIXS spectrometer based on the Rowland circle geometry (Shvyd’ko *et al.*, 2013 ▸; Rueff *et al.*, 2015 ▸; Moretti Sala *et al.*, 2018 ▸). The RIXS spectrometer is installed on a four-circle kappa diffractometer that provides a wide range of the chi (χ) motion of the sample. The advantages of using a kappa-type diffractometer will be discussed in more detail in Section 4. The photon flux at the Ir *L*_3_-edge is 1.5 × 10^11^ photons s^−1^ after the DCM and 7.1 × 10^8^ photons s^−1^ after the HRM. The flux was measured at a storage ring current of 400 mA using a calibrated 300 µm-thick silicon photodiode with corrections including detector efficiency (Owen *et al.*, 2009 ▸). Although the beam flux limited by the 3 GeV ring is two orders of magnitude lower compared with that at Sector 27 of the Advanced Photon Source (Shvyd’ko *et al.*, 2013 ▸; Gog *et al.*, 2018 ▸), the count rate at the detector is partially compensated by the larger solid angle of acceptance of the 1 m Rowland circle geometry in our RIXS setup. The accessible absorption edges with current in-stock analyzers and channel-cuts are tabulated in Table 1 ▸ with the estimated total energy resolutions, which take into account the contributions from the incident energy bandwidth, Darwin width, geometrical broadening, finite beam-size effect and Johann aberration.

## 3. Focusing

A pair of KB mirrors from Thales SESO focuses the monochromatic beam after the HRM. The horizontal focusing mirror (HFM) has a platinum coating with a thickness of 50 nm and a tangential slope error of 2 µrad RMS. The vertical focusing mirror (VFM), on the other hand, has a unique coating consisting of platinum and palladium, each with a thickness of 50 nm, separated into different areas and has a tangential slope error of 1 µrad RMS. The platinum-coated area is typically used at the Ir *L*_3_-edge, whereas the palladium-coated area is used for lower energies to achieve higher reflection efficiency.

The incident beam with dimensions 3.5 mm × 1.0 mm (H × V) is first reflected by the 800 mm-long HFM at an incidence angle of 5 mrad, and then by the 150 mm-long VFM at an incidence angle of 3 mrad. The KB mirrors are capable of accepting a beam size of 4 mm × 0.5 mm, which can fully cover the profile in the horizontal direction but only half of the beam in the vertical direction. Due to limited space inside the hutch, the vertical mirror had to be designed to be shorter than necessary to accept the full vertical profile of X-rays. The X-rays that spill over the vertical mirror are blocked by a four-blade slit (HS2) after the mirrors. The total efficiency of the KB mirrors is approximately 40%, primarily due to the short VFM (50%) and the reflection efficiency of platinum coatings (∼80%).

**Figure 2:**
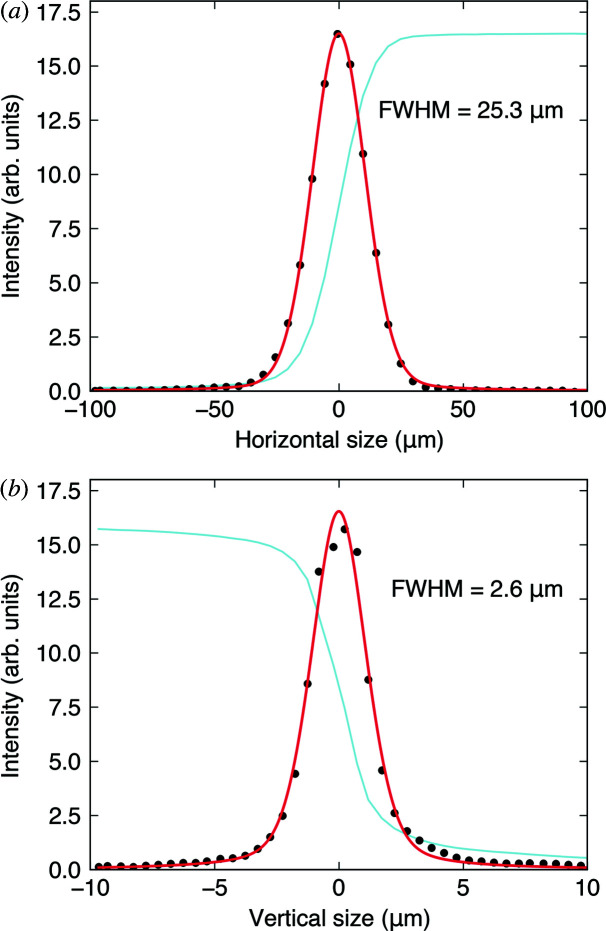
(*a*) Measured horizontal size of the focused beam by KB mirrors at the sample position. (*b*) Measured vertical size of the focused beam by KB mirrors at the sample position. The beam sizes are estimated by fitting (red curves) the derivatives (black dots) of knife-edge scans (light blue curves).

Fig. 2 ▸ shows the 25.3 µm × 2.6 µm (H × V) focused beam size at the sample position, measured by knife-edge scans. The beam size is estimated by fitting the derivative of the knife-edge scans in both the horizontal and the vertical directions using a pseudo-Voigt function. The measured beam size is close to the theoretically estimated size of 28.4 µm × 1.9 µm calculated only by the demagnification factors from the focal lengths. The focal lengths of the HFM and the VFM are 1975 mm and 1400 mm located at 34.725 m and 36.7 m from the source, respectively. The source size, 500 µm × 50 µm (H × V), then provides the theoretical values for the focused beam size at the sample position, 28.4 µm × 1.9 µm. A vertical beam size of 2.6 µm is notably smaller than those of other hard X-ray RIXS beamlines (Shvyd’ko *et al.*, 2013 ▸; Moretti Sala *et al.*, 2018 ▸), which prevents a potential broadening of energy resolution by a finite beam size.

## 4. Spectrometer

**Figure 3:**
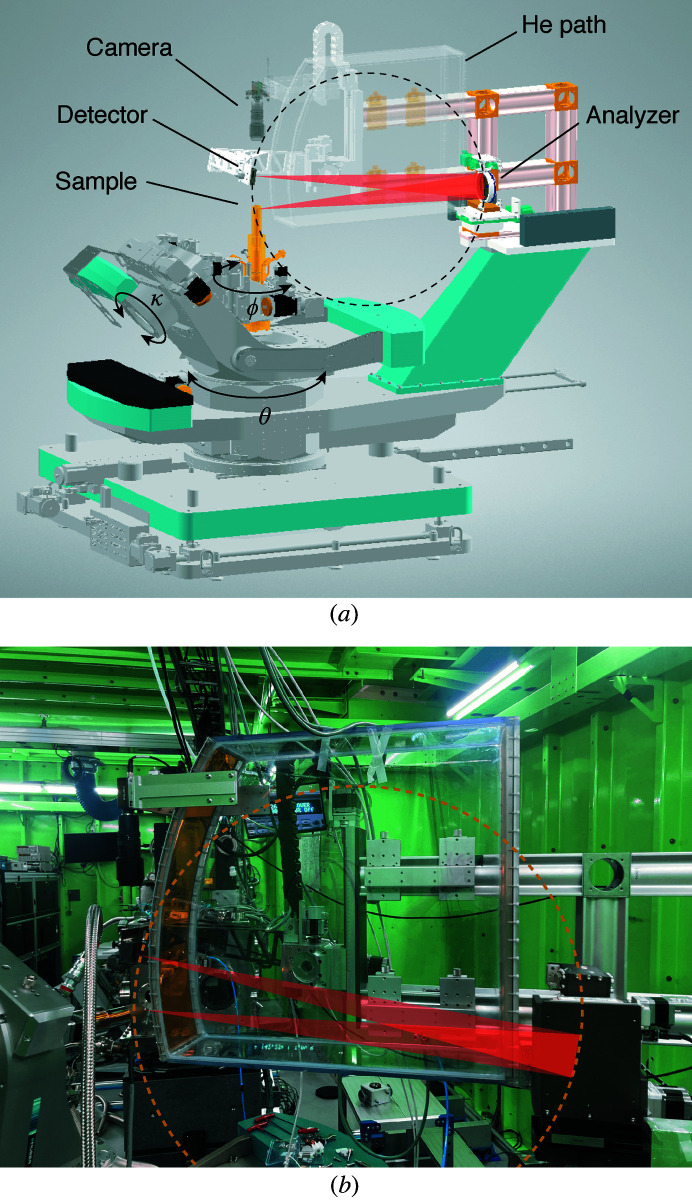
(*a*) Drawing of a RIXS spectrometer installed on a four-circle kappa diffractometer. A sample is mounted on the cryostat and manipulated by micro-motion stages. Scattered X-rays are reflected and focused onto the detector following the Rowland circle geometry. A flight path filled with helium gas is installed on the X-ray path to minimize air scattering. A sample camera is installed above the sample and the detector. Arrows indicate the rotations that are used to implement the chi (χ) rotation. (*b*) Photograph of the RIXS spectrometer installed at the 1C beamline of PLS-II.

For the energy and momentum analyses, we installed a RIXS spectrometer based on the Rowland circle geometry on a four-circle kappa diffractometer as shown in Fig. 3 ▸. A kappa-type diffractometer implements the chi (χ) rotation in the Euler geometry through the combination of kappa (κ), theta (θ) and phi (ϕ) rotations. The advantage of using a kappa-type diffractometer is that it provides a wide range of the χ rotation without a χ cradle, which interferes with the detector located right above the sample in the Rowland circle geometry [Figs. 3 ▸ and 4 ▸(*a*)]. We note that most RIXS spectrometers based on the Eulerian diffractometer employ an arc segment for the χ rotation with a limited range (Shvyd’ko *et al.*, 2013 ▸; Moretti Sala *et al.*, 2018 ▸). On the contrary, the κ angle of our diffractometer (50°) guarantees the χ range ±100°. Also, in a horizontal scattering geometry where RIXS measurements are conducted, χ is at 90° and the ϕ rotation provides a full azimuthal angle range.

These large degrees of freedom in rotations of the sample allow us to explore more Brillouin zones and measure in a more extreme scattering geometry. It has been shown in RIXS measurements of a square-lattice iridate that RIXS in normal or grazing incident geometry – where the incident angle is close to 0° or 90°, respectively – enhances or suppresses one of the polarization components (Porras *et al.*, 2019 ▸; Bertinshaw *et al.*, 2020 ▸). However, with a limited χ range, it is hard to control the incident angle close to 0° or 90°. For example, *Q* = (3 2 28.2) was used to make a grazing incidence geometry in previous work (Porras *et al.*, 2019 ▸), where χ ≃ 6.7° and the incident angle is maintained around 14.5°. In contrast, with our diffractometer, an incdient angle below 1° can be achieved with *Q* = (5 2 21.5) which requires a high value of χ ≃ 25°. Also, it would be useful to carry out thin-film measurements which require a grazing incident geometry to increase the footprint of the X-rays.

**Figure 4:**
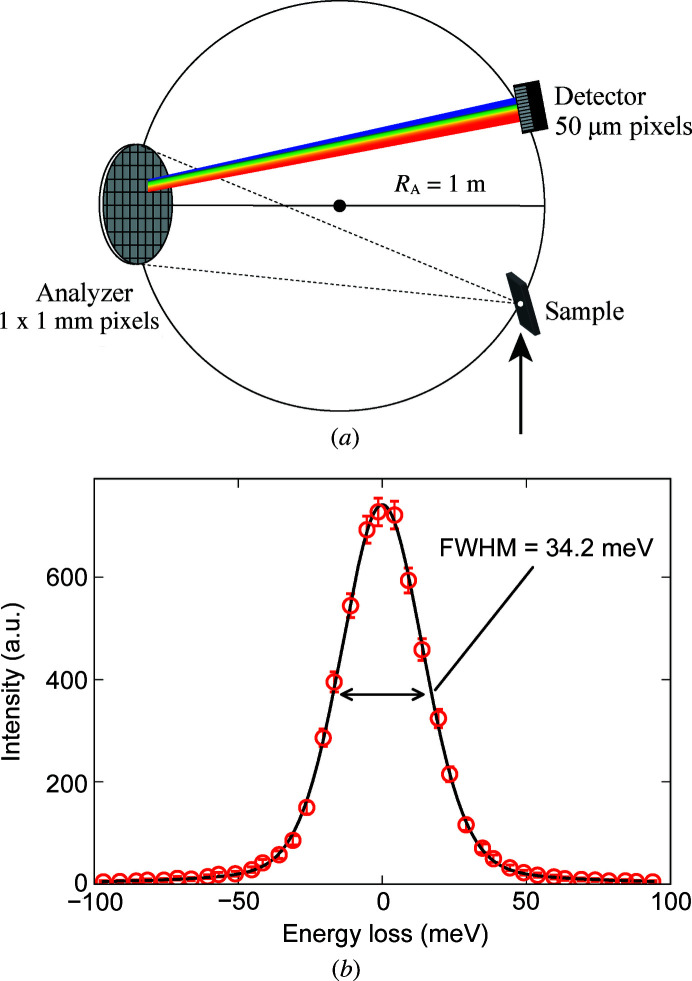
(*a*) Schematic of the RIXS spectrometer. The sample, analyzer and detector are placed on the Rowland circle so that the energies of the scattered X-rays can be analyzed by their position on the detector. The large arrow under the sample indicates the incident X-rays. (*b*) Total energy resolution of the RIXS spectrometer measured by a reference elastic scatterer, twelve layers of 3M Magic Scotch tape. The red dots are the measured points and the black curve is the fitting with a pseudo-Voigt function, which gives 34.2 meV full width at half-maximum (FWHM).

The introduction of the Rowland circle geometry (Johann, 1931 ▸), the advances in fabrication technology for diced spherical analyzers (Masciovecchio *et al.*, 1996 ▸; Verbeni *et al.*, 2005 ▸; Said *et al.*, 2011 ▸) and the use of a position-sensitive detector (Huotari *et al.*, 2005 ▸) significantly enhance the energy resolution and the photon counts simultaneously (Shvyd’ko *et al.*, 2013 ▸). In this geometry, the scattered photons from the sample are analyzed by a diced spherical analyzer and focused onto a position-sensitive detector [Fig. 4 ▸(*a*)]. The analyzer and the detector are placed on the Rowland circle with a radius *R*_A_ = 1 m [Fig. 4 ▸(*a*)] and a flight path filled with helium gas is installed on the beam path to reduce air scattering and maximize photon counts (Fig. 3 ▸). At the Ir *L*_3_-edge, an Si(844) diced spherical analyzer is used made of a 3 mm-thick Si(844) wafer and glued on top of a concave glass to create a curvature radius of 1 m. It has a 10 cm diameter and has square pixels (or dices) with dimensions 1 mm × 1 mm. A manual iris in front of the analyzer allows for continuous adjustment of its active area, enabling improvement of the momentum resolution in exchange for photon counts. The flat surface of the dices with a 1 mm vertical size accepts a finite energy window, 0.84 eV, of the scattered X-rays from the sample since the incident angle to the analyzer varies at each vertical position of a dice. The analyzer then disperses these X-rays according to their energies following Bragg’s law and maps them onto a position-sensitive Dectris MYTHEN2 detector which has 640 pixels with a size of 50 µm [Fig. 4 ▸(*a*)]. The use of this position-sensitive detector dramatically suppresses the geometrical broadening contribution to the energy resolution of the spectrometer (Huotari *et al.*, 2005 ▸). The resulting geometrical broadening Δ*E*_g_ of the spectrometer is determined by the pixel size of the detector Δ*d*_D_ (Shvyd’ko *et al.*, 2013 ▸) as

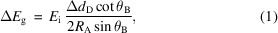

where θ_B_ is the Bragg angle of the analyzer reflection. This geometrical broadening of our spectrometer (*R*_A_ = 1 m and Δ*d*_D_ = 50 µm) at the Ir *L*_3_-edge is calculated as Δ*E*_g_ = 20.94 meV.

The total energy resolution of our RIXS spectrometer was determined by measuring 3M Magic Scotch tape as an elastic reference scatterer and fitting the results with a pseudo-Voigt function as shown in Fig. 4 ▸(*b*). Our analysis shows a total resolution of 34.2 meV, which is in close agreement with the theoretically calculated value of 34.0 meV, as presented in Table 1 ▸. In order to calculate the total energy resolution at various edges, we considered five different contributions: (i) the energy bandpass of incident X-rays (Δ*E*_i_), (ii) the intrinsic resolution (Darwin width) of the analyzer (Δ*E*_a_), (iii) the geometrical contribution defined in equation (2) (Δ*E*_g_), (iv) the finite beam-size effect (Δ*E*_s_) and (v) the Johann aberration (Δ*E*_J_).

The broadening caused by the finite beam size was calculated by estimating the Bragg angle variation due to the vertical beam size at the sample position, as described by (Moretti Sala *et al.* 2018 ▸) 

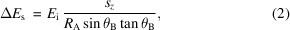

where *s*_*z*_ is the vertical beam size at the sample position. We did not consider the horizontal beam size as it results in a negligible amount of broadening. The contribution from the Johann aberration, which arises from the deviation of the curvature of the spherical analyzer from the ideal angle for focusing the scattered photons, was estimated by the weighted average of the Bragg angle variation across the circular analyzer, as given by (Moretti Sala *et al.*, 2018 ▸)

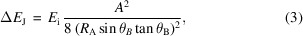

where *A* is the analyzer iris radius. The energy resolutions of the various accessible absorption edges, using our in-stock optical components, are summarized in Table 1 ▸. We note that the contribution from the Johann aberration (Δ*E*_J_) usually dominates the total energy resolution, except for the Ir *L*_3_-edge where the reflection is close to back-scattering. However, the Johann aberration can be mitigated by reducing the active analyzer area. For the W *L*_3_-edge, reducing the diameter of the analyzer iris to half its original size results in a reduction of the Johann contribution to 79 meV, leading to a significantly improved total energy resolution of 129 meV.

**Figure 5:**
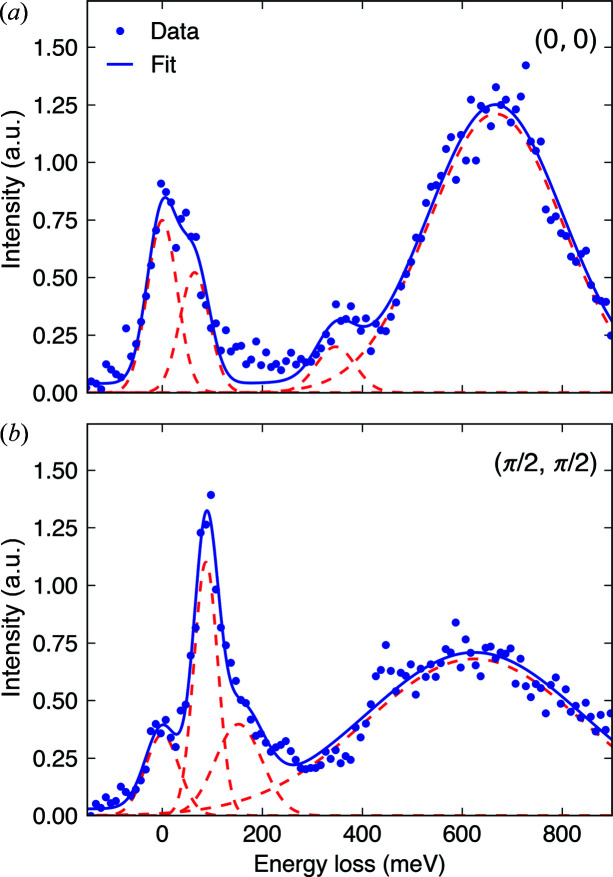
(*a*) RIXS spectrum of Sr_2_IrO_4_ at *q* = (0, 0). The peak at 40 meV corresponds to the out-of-plane magnon mode, the peak around 350 meV to spin-orbit excitons and the broad continuum around 600 meV to *dd*-excitations within *t*_2g_ orbitals. (*b*) RIXS spectrum of Sr_2_IrO_4_ at *q* = (π/2, π/2). The peak around 100 meV corresponds to single-magnon excitations, the broader peak around 150 meV corresponds to multimagnons and the broad continuum around 600 meV to *dd*-excitations within *t*_2g_ orbitals. The spectra shown in (*a*) and (*b*) are collected at 80 K over 1 h each. Gaussian functions are used to fit all the peaks, displayed as red-dashed curves. The sum of all fit functions is drawn as a blue curve.

Fig. 5 ▸ shows the RIXS spectra of Sr_2_IrO_4_ measured at (0, 0) and (π/2, π/2) using our endstation. Both are measured at *L* = 32.5 to minimize elastic scattering with 2θ ≃ 90°. We note that the low-energy excitations of Sr_2_IrO_4_ are almost two-dimensional and independent of a momentum transfer along the *c* axis (Kim *et al.*, 2012*a* ▸; Ishii *et al.*, 2011 ▸). The analyzer iris was fully open to 9 cm diameter which produces a momentum resolution of ∼0.32 r.l.u. (in-plane reciprocal lattice units) in the *H* or *K* direction. The (0, 0) spectrum in Fig. 5 ▸(*a*) shows the out-of-plane magnon mode at 40 meV (Pincini *et al.*, 2017 ▸; Porras *et al.*, 2019 ▸) clearly resolved from the elastic peak. Other features such as the spin–orbit excitons at 350 meV and *dd*-excitations around 600 meV are also well reproduced (Kim *et al.*, 2012*a* ▸, 2014 ▸; Gretarsson *et al.*, 2016 ▸; Bertinshaw *et al.*, 2020 ▸). The (π/2, π/2) spectrum in Fig. 5 ▸(*b*) exhibits a resolution-limited single magnon around 100 meV with a count rate of approximately 45 counts per minute at a ring current of 400 mA. Overall, the RIXS spectra in Fig. 5 ▸ are in excellent agreement with previously reported results, demonstrating the performance of the RIXS spectrometer constructed at the 1C beamline of the PLS-II.

## 5. X-ray diffuse scattering and diffraction

**Figure 6:**
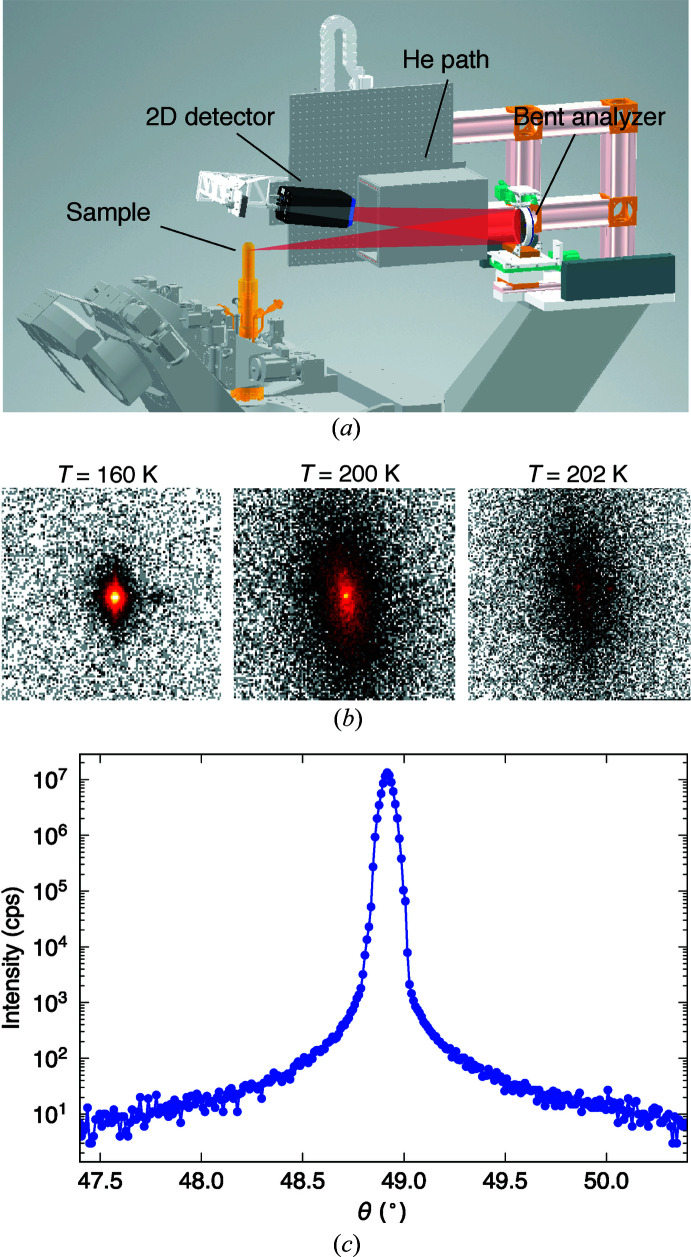
(*a*) Drawing of an X-ray diffuse scattering setup. This setup is a reconfiguration from the original RIXS spectrometer shown in Fig. 3 ▸(*a*) by incorporating a bent analyzer in place of a diced spherical analyzer. Additionally, a 2D area detector is placed before a position-sensitive detector, resulting in a slightly altered flight path with reduced dimensions. (*b*) Raw 2D detector images of a charge density wave (CDW) peak in TiSe_2_, taken at three different temperatures (*T* = 160, 200, 202 K) across the critical temperature *T*_c_ = 200 K. The measurements were performed at the wavevector (0.5 0.5 4.5). An Si(844)-bent spherical analyzer was used with 11.215 keV incident X-rays. With this incident energy, the bent analyzer with a 10 cm diameter accepts a circular area in momentum space with a diameter of 0.3 Å^−1^ in a single shot which corresponds to about 0.17 r.l.u. *H* (or *K*) for TiSe_2_. (*c*) θ–2θ rocking curve measurement of the (1 0 5) charge peak of TiSe_2_ at room temperature.

The endstation can be easily reconfigured for measurements in which only energy-integrated intensities are needed, such as X-ray diffuse scattering and diffraction [Fig. 6 ▸(*a*)]. X-ray diffuse scattering measures short-range correlations between ionic positions or spins from energy-integrated signals, and thus is useful to reveal the nature of microscopic interactions between them. For example, X-ray thermal diffuse scattering can determine the phonon dispersion relations (Xu & Chiang, 2005 ▸) which reveal how atoms interact with each other in solids, and X-ray magnetic diffuse scattering can provide important insights into the anisotropy of spin interactions (Chun *et al.*, 2015 ▸).

However, high efficiency and low noise level are essential for this technique since it measures small quasi-elastic signals that are diffuse in momentum space. To this end, our setup utilizes a bent spherical analyzer and the DECTRIS EIGER2 R 1M 2D area detector as shown in Fig. 6 ▸(*a*). The bent analyzer replaces the diced spherical analyzer and the 2D detector is placed before the position-sensitive detector. Unlike the diced analyzer, the bent analyzer preserves the spatial information of scatterings and maps them onto 2D images on the 2D detector, which can then be converted to momentum space. Therefore, this setup can efficiently measure diffuse signals for a finite area in momentum space in a single measurement. In addition, the bent analyzer with a broad energy bandwidth (∼2.7 eV) can accept 100× more photons compared with the diced spherical analyzer.

Fig. 6 ▸(*b*) shows raw images of a charge density wave (CDW) peak of TiSe_2_ taken by the 2D area detector over 10 s. Despite the short measuring time, our setup successfully measures diffuse signals around the CDW peak across the critical temperature (*T*_c_ = 200 K). Furthermore, the reflection geometry along with a finite energy bandwidth of the analyzer effectively reduce the noise level in measurements, as demonstrated by the impressive signal-to-noise ratio (∼10^6^) observed in the θ–2θ rocking curve of a charge Bragg peak of TiSe_2_ in Fig. 6 ▸(*c*).

## 6. Conclusions

We have described and demonstrated the performance of the RIXS endstation installed at the 1C beamline of PLS-II. The main components of the endstation include a high-resolution channel-cut monochromator, a pair of focusing KB mirrors and an RIXS spectrometer. The use of a kappa-type diffractometer for the spectrometer provides a wide range of accessible scattering geometries. We achieved a total energy resolution of 34.2 meV at the Ir *L*_3_-edge which is close to the calculated value of 34.0 meV. Our measurement on Sr_2_IrO_4_ reproduces all the features observed in prior works, and thus demonstrates that a scan over a 1 eV energy range can be acquired within an hour with good statistics. Further, our X-ray diffuse scattering setup in a reflection geometry demonstrates acquiring high-quality diffuse scattering images in ∼10 s. 

## Figures and Tables

**Table 1:** Current accessible absorption edges and theoretically estimated total energy resolutions (Δ*E*_t_) Total energy resolution is estimated by considering the incident energy bandpass (Δ*E*_i_), Darwin width (Δ*E*_a_), geometrical broadening (Δ*E*_g_), finite beam-size effect (Δ*E*_s_) and Johann aberration (Δ*E*_J_). The radius of the analyzer iris is set to full size (*A* = 45 mm) and the radius of the Rowland circle is fixed to *R* = 1 m.

	Ir *L*_3_	Ta *L*_3_	W *L*_3_	Nd *L*_3_	Cu *K*
*E*_i_ (keV)	11.215	9.881	10.207	6.208	8.9805
Reflection	Si(844)	Si(660)	Si(660)	Si(333)	Si(553)
θ_B_ (°)	85.73	78.58	71.61	72.82	77.5
Δ*E*_i_ (meV)	15.8	32.85	50.44	52.94	33.15
Δ*E*_a_ (meV)	14.57	23.02	23.73	53.25	21.56
Δ*E*_g_ (meV)	20.94	49.88	84.86	47.98	49.78
Δ*E*_s_ (meV)	2.18	5.29	9.3	5.22	5.3
Δ*E*_J_ (meV)	15.74	98.05	257.15	137.1	106.49
Δ*E*_t_ (meV)	34.0	117.2	276.6	163.6	124.1
